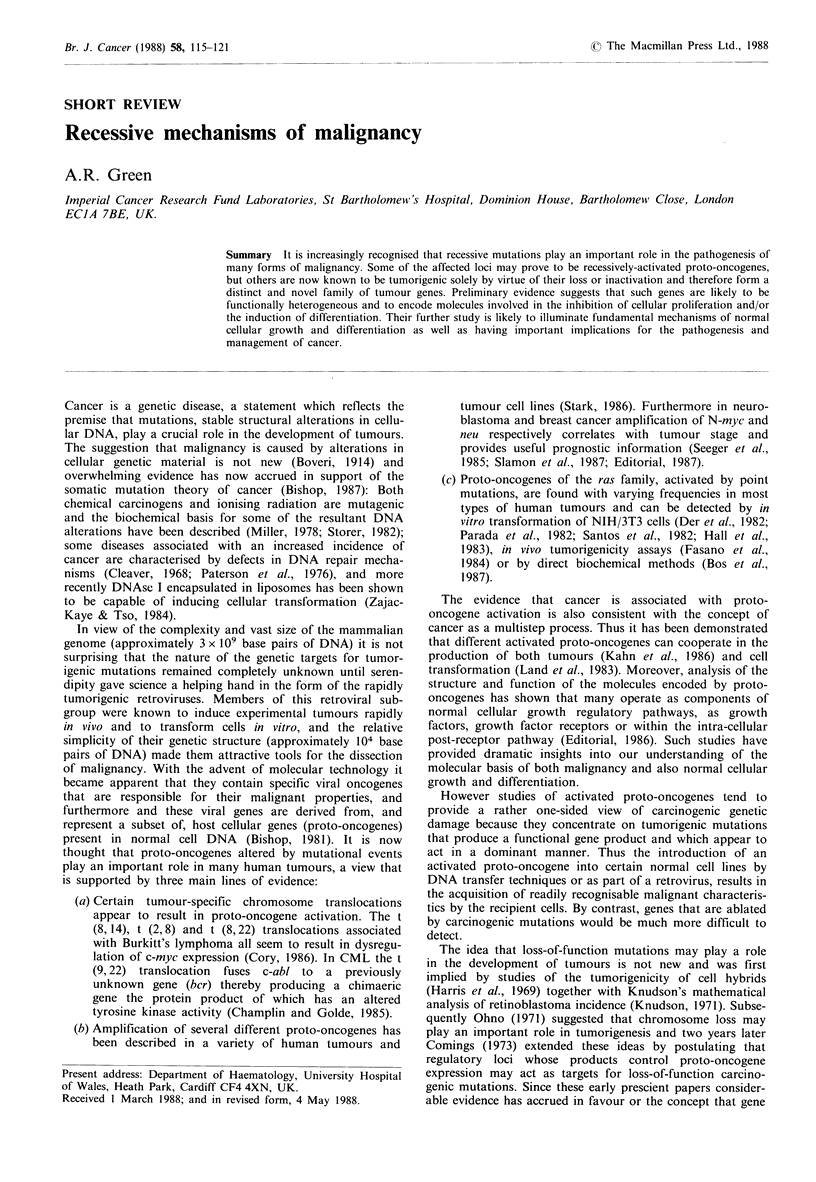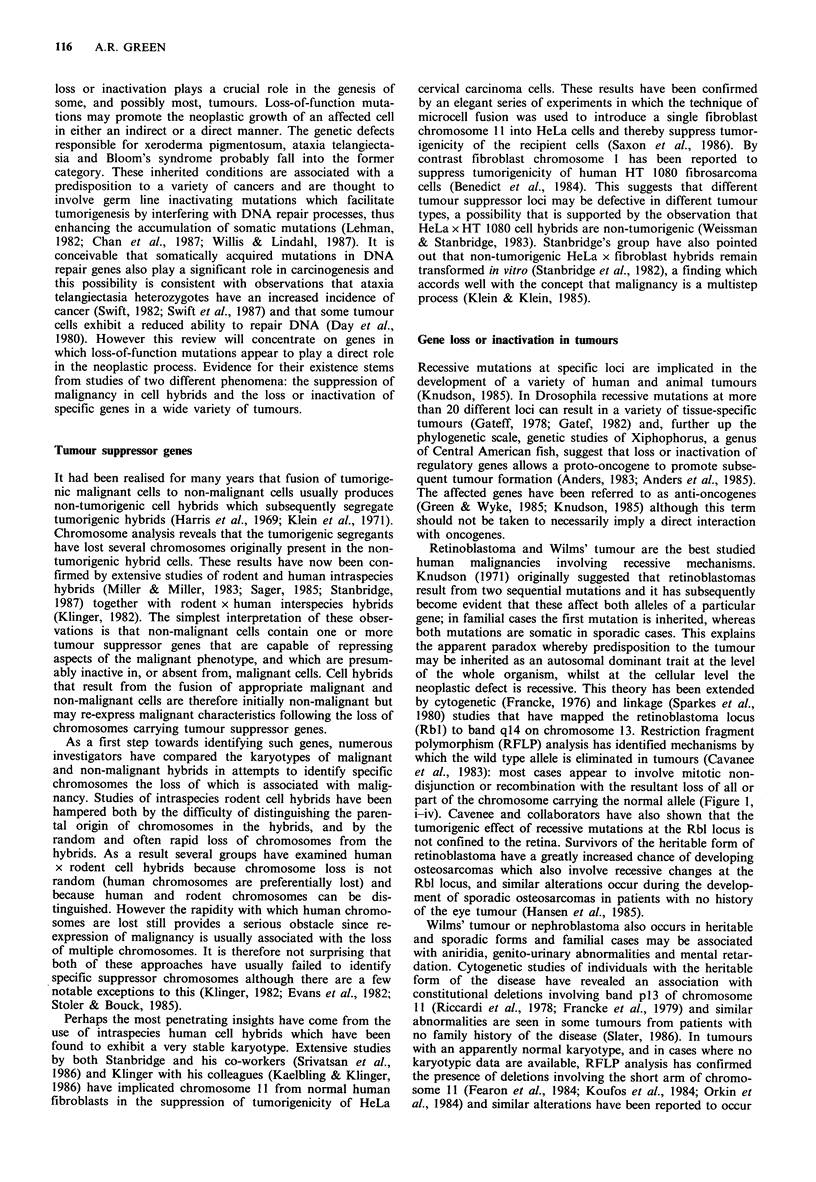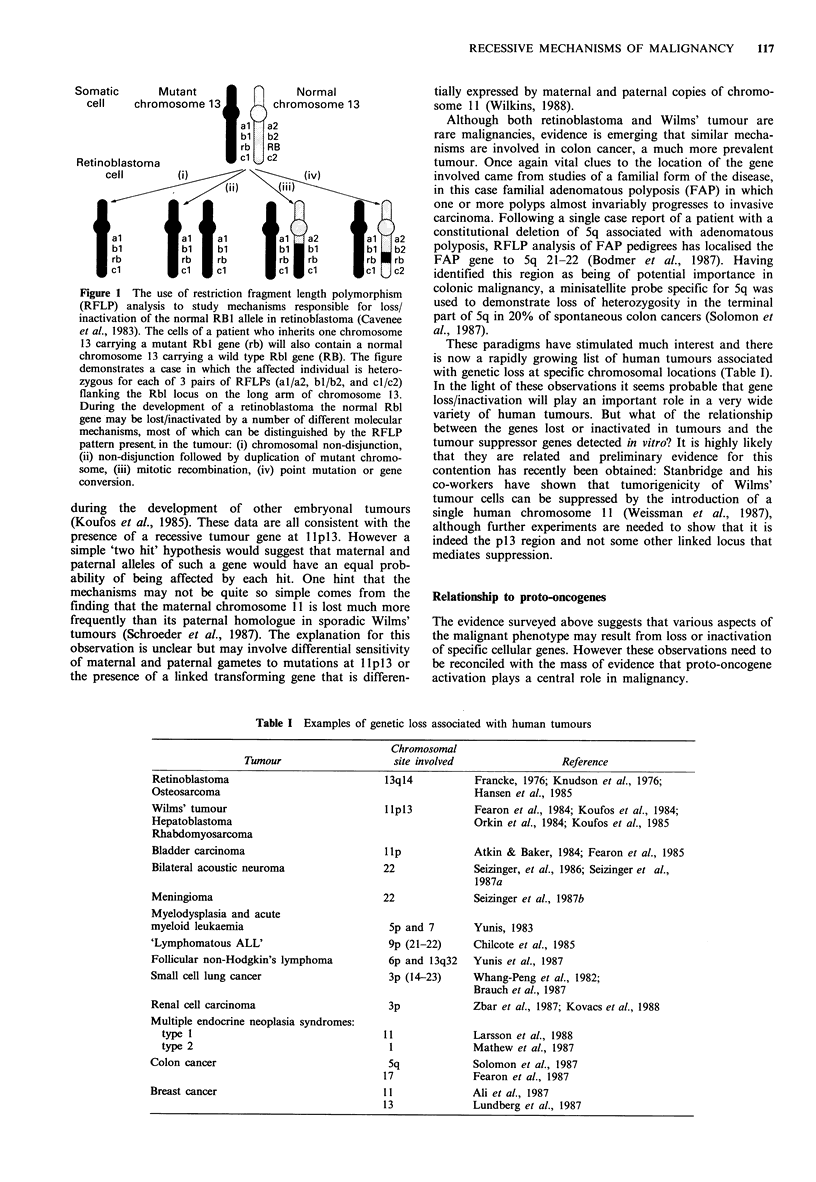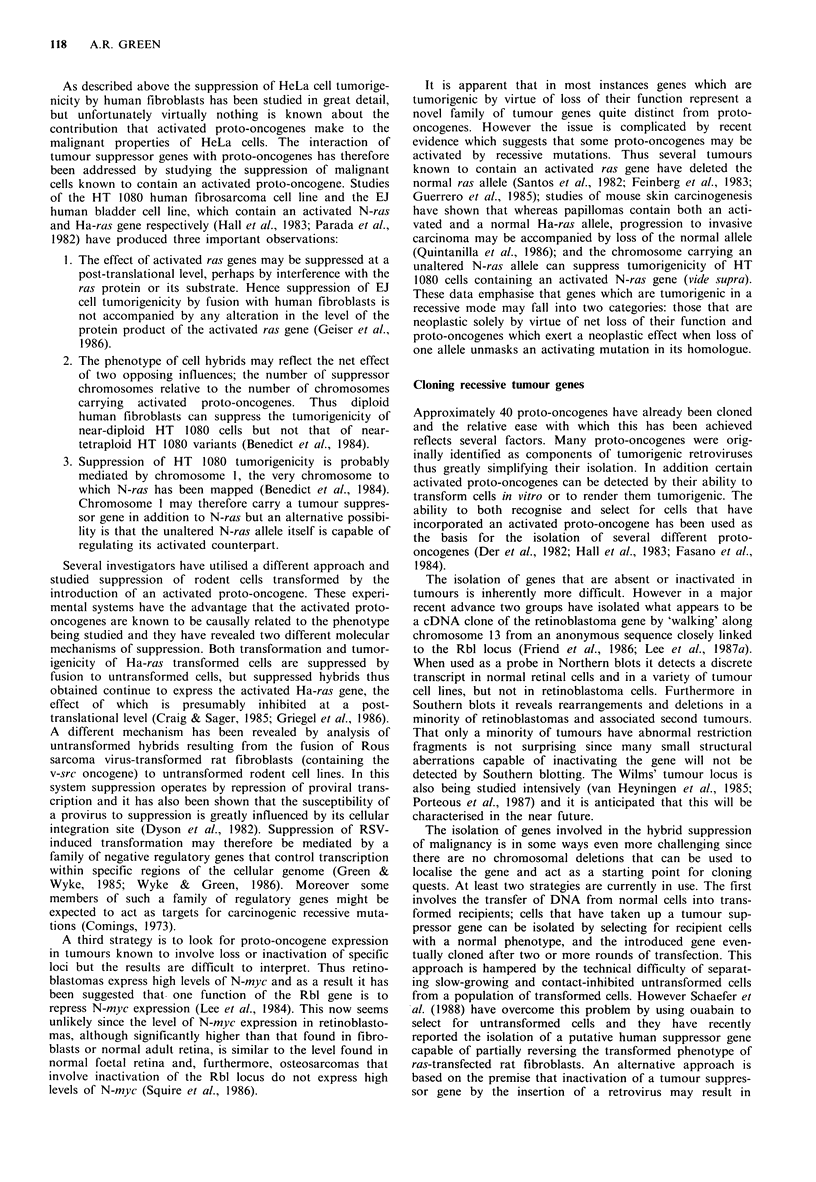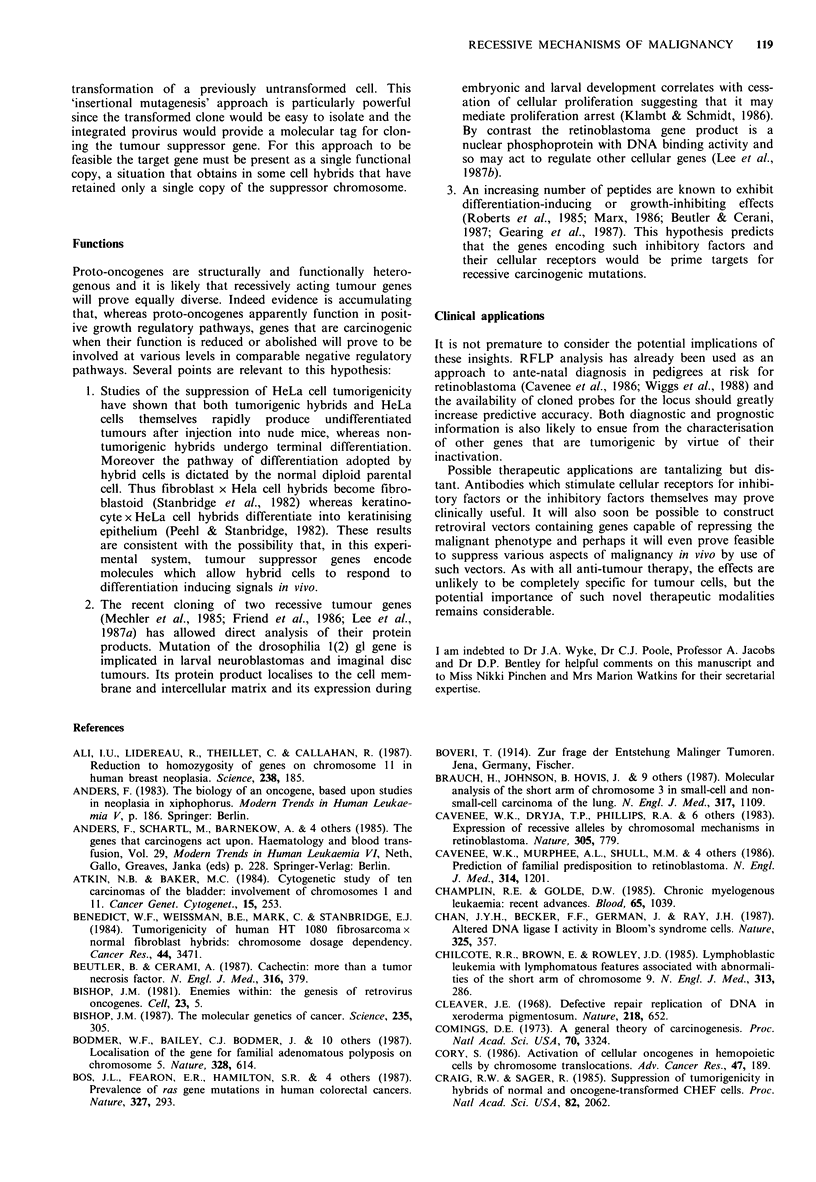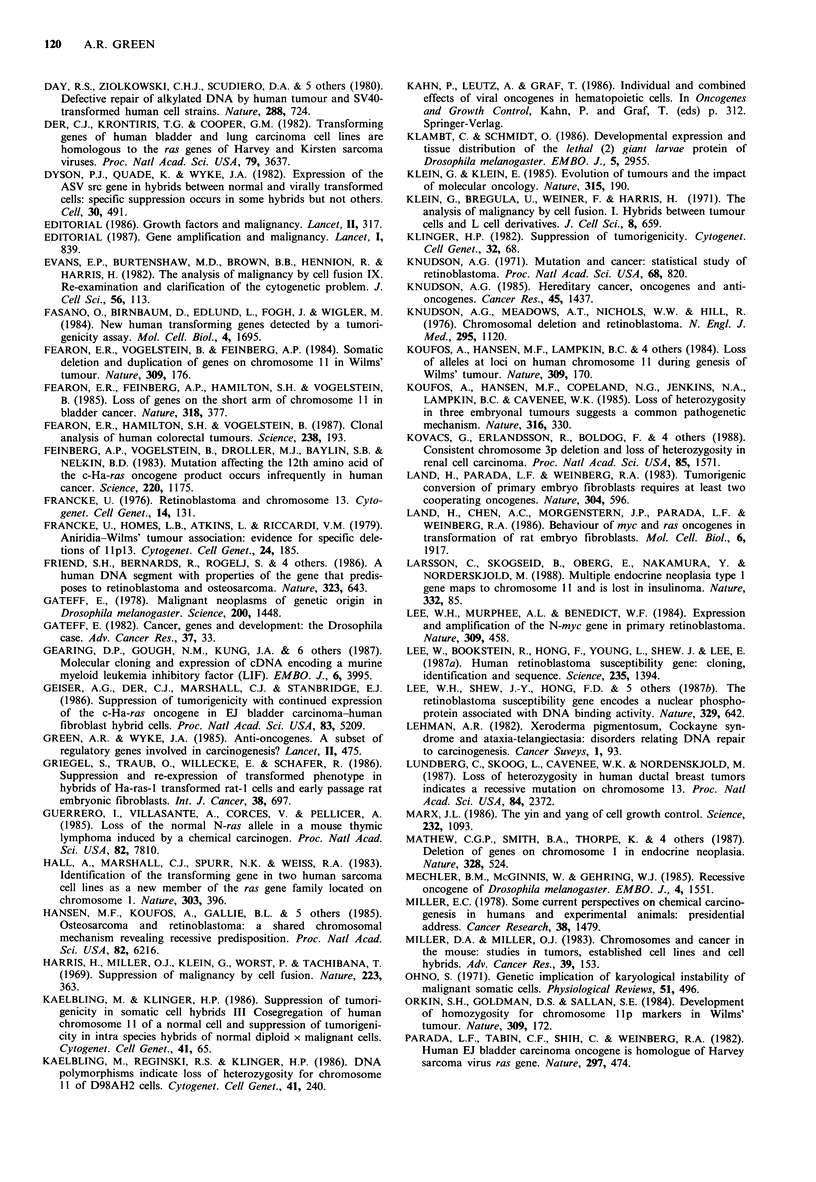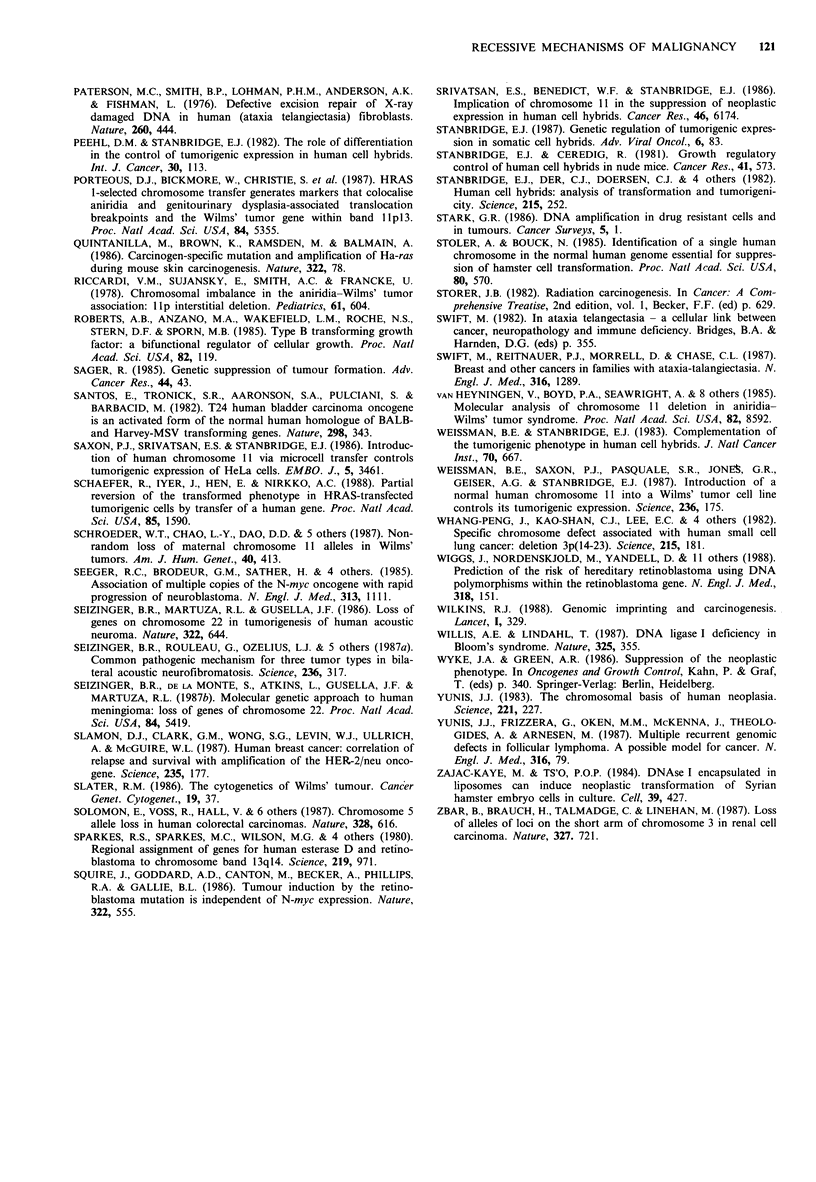# Recessive mechanisms of malignancy.

**DOI:** 10.1038/bjc.1988.176

**Published:** 1988-08

**Authors:** A. R. Green

**Affiliations:** Imperial Cancer Research Fund Laboratories, St Bartholomew's Hospital, London, UK.

## Abstract

It is increasingly recognised that recessive mutations play an important role in the pathogenesis of many forms of malignancy. Some of the affected loci may prove to be recessively-activated proto-oncogenes, but others are now known to be tumorigenic solely by virtue of their loss or inactivation and therefore form a distinct and novel family of tumour genes. Preliminary evidence suggests that such genes are likely to be functionally heterogeneous and to encode molecules involved in the inhibition of cellular proliferation and/or the induction of differentiation. Their further study is likely to illuminate fundamental mechanisms of normal cellular growth and differentiation as well as having important implications for the pathogenesis and management of cancer.


Br. J. Cancer (1988) 58, 115–121 © The Macmillan Press Ltd., 1988

SHORT REVIEW

### Recessive mechanisms of malignancy

A.R. Green

*Imperial Cancer Research Fund Laboratories, St Bartholomew's Hospital, Dominion House, Bartholomew Close, London EC1A 7BE, UK.*

**Summary** It is increasingly recognised that recessive mutations play an important role in the pathogenesis of many forms of malignancy. Some of the affected loci may prove to be recessively-activated proto-oncogenes, but others are now known to be tumorigenic solely by virtue of their loss or inactivation and therefore form a distinct and novel family of tumour genes. Preliminary evidence suggests that such genes are likely to be functionally heterogeneous and to encode molecules involved in the inhibition of cellular proliferation and/or the induction of differentiation. Their further study is likely to illuminate fundamental mechanisms of normal cellular growth and differentiation as well as having important implications for the pathogenesis and management of cancer.

Cancer is a genetic disease, a statement which reflects the premise that mutations, stable structural alterations in cellular DNA, play a crucial role in the development of tumours. The suggestion that malignancy is caused by alterations in cellular genetic material is not new (Boveri, 1914) and overwhelming evidence has now accrued in support of the somatic mutation theory of cancer (Bishop, 1987): Both chemical carcinogens and ionising radiation are mutagenic and the biochemical basis for some of the resultant DNA alterations have been described (Miller, 1978; Storer, 1982); some diseases associated with an increased incidence of cancer are characterised by defects in DNA repair mechanisms (Cleaver, 1968; Paterson *et al.*, 1976), and more recently DNAse I encapsulated in liposomes has been shown to be capable of inducing cellular transformation (Zajac-Kaye & Tso, 1984).

In view of the complexity and vast size of the mammalian genome (approximately $3\times10^9$ base pairs of DNA) it is not surprising that the nature of the genetic targets for tumorigenic mutations remained completely unknown until serendipity gave science a helping hand in the form of the rapidly tumorigenic retroviruses. Members of this retroviral subgroup were known to induce experimental tumours rapidly *in vivo* and to transform cells *in vitro*, and the relative simplicity of their genetic structure (approximately $10^4$ base pairs of DNA) made them attractive tools for the dissection of malignancy. With the advent of molecular technology it became apparent that they contain specific viral oncogenes that are responsible for their malignant properties, and furthermore and these viral genes are derived from, and represent a subset of, host cellular genes (proto-oncogenes) present in normal cell DNA (Bishop, 1981). It is now thought that proto-oncogenes altered by mutational events play an important role in many human tumours, a view that is supported by three main lines of evidence:

(*a*) Certain tumour-specific chromosome translocations appear to result in proto-oncogene activation. The t (8,14), t (2,8) and t (8,22) translocations associated with Burkitt's lymphoma all seem to result in dysregulation of c-*myc* expression (Cory, 1986). In CML the t (9,22) translocation fuses c-*abl* to a previously unknown gene (*bcr*) thereby producing a chimaeric gene the protein product of which has an altered tyrosine kinase activity (Champlin and Golde, 1985).

(*b*) Amplification of several different proto-oncogenes has been described in a variety of human tumours and tumour cell lines (Stark, 1986). Furthermore in neuroblastoma and breast cancer amplification of N-*myc* and *neu* respectively correlates with tumour stage and provides useful prognostic information (Seeger *et al.*, 1985; Slamon *et al.*, 1987; Editorial, 1987).

(*c*) Proto-oncogenes of the *ras* family, activated by point mutations, are found with varying frequencies in most types of human tumours and can be detected by *in vitro* transformation of NIH/3T3 cells (Der *et al.*, 1982; Parada *et al.*, 1982; Santos *et al.*, 1982; Hall *et al.*, 1983), *in vivo* tumorigenicity assays (Fasano *et al.*, 1984) or by direct biochemical methods (Bos *et al.*, 1987).

The evidence that cancer is associated with proto-oncogene activation is also consistent with the concept of cancer as a multistep process. Thus it has been demonstrated that different activated proto-oncogenes can cooperate in the production of both tumours (Kahn *et al.*, 1986) and cell transformation (Land *et al.*, 1983). Moreover, analysis of the structure and function of the molecules encoded by proto-oncogenes has shown that many operate as components of normal cellular growth regulatory pathways, as growth factors, growth factor receptors or within the intra-cellular post-receptor pathway (Editorial, 1986). Such studies have provided dramatic insights into our understanding of the molecular basis of both malignancy and also normal cellular growth and differentiation.

However studies of activated proto-oncogenes tend to provide a rather one-sided view of carcinogenic genetic damage because they concentrate on tumorigenic mutations that produce a functional gene product and which appear to act in a dominant manner. Thus the introduction of an activated proto-oncogene into certain normal cell lines by DNA transfer techniques or as part of a retrovirus, results in the acquisition of readily recognisable malignant characteristics by the recipient cells. By contrast, genes that are ablated by carcinogenic mutations would be much more difficult to detect.

The idea that loss-of-function mutations may play a role in the development of tumours is not new and was first implied by studies of the tumorigenicity of cell hybrids (Harris *et al.*, 1969) together with Knudson's mathematical analysis of retinoblastoma incidence (Knudson, 1971). Subsequently Ohno (1971) suggested that chromosome loss may play an important role in tumorigenesis and two years later Comings (1973) extended these ideas by postulating that regulatory loci whose products control proto-oncogene expression may act as targets for loss-of-function carcinogenic mutations. Since these early prescient papers considerable evidence has accrued in favour or the concept that gene

Present address: Department of Haematology, University Hospital of Wales, Heath Park, Cardiff CF4 4XN, UK.
Received 1 March 1988; and in revised form, 4 May 1988.

loss or inactivation plays a crucial role in the genesis of some, and possibly most, tumours. Loss-of-function mutations may promote the neoplastic growth of an affected cell in either an indirect or a direct manner. The genetic defects responsible for xeroderma pigmentosum, ataxia telangiectasia and Bloom's syndrome probably fall into the former category. These inherited conditions are associated with a predisposition to a variety of cancers and are thought to involve germ line inactivating mutations which facilitate tumorigenesis by interfering with DNA repair processes, thus enhancing the accumulation of somatic mutations (Lehman, 1982; Chan *et al.*, 1987; Willis & Lindahl, 1987). It is conceivable that somatically acquired mutations in DNA repair genes also play a significant role in carcinogenesis and this possibility is consistent with observations that ataxia telangiectasia heterozygotes have an increased incidence of cancer (Swift, 1982; Swift *et al.*, 1987) and that some tumour cells exhibit a reduced ability to repair DNA (Day *et al.*, 1980). However this review will concentrate on genes in which loss-of-function mutations appear to play a direct role in the neoplastic process. Evidence for their existence stems from studies of two different phenomena: the suppression of malignancy in cell hybrids and the loss or inactivation of specific genes in a wide variety of tumours.

### Tumour suppressor genes

It had been realised for many years that fusion of tumorigenic malignant cells to non-malignant cells usually produces non-tumorigenic cell hybrids which subsequently segregate tumorigenic hybrids (Harris *et al.*, 1969; Klein *et al.*, 1971). Chromosome analysis reveals that the tumorigenic segregants have lost several chromosomes originally present in the non-tumorigenic hybrid cells. These results have now been confirmed by extensive studies of rodent and human intraspecies hybrids (Miller & Miller, 1983; Sager, 1985; Stanbridge, 1987) together with rodent × human interspecies hybrids (Klinger, 1982). The simplest interpretation of these observations is that non-malignant cells contain one or more tumour suppressor genes that are capable of repressing aspects of the malignant phenotype, and which are presumably inactive in, or absent from, malignant cells. Cell hybrids that result from the fusion of appropriate malignant and non-malignant cells are therefore initially non-malignant but may re-express malignant characteristics following the loss of chromosomes carrying tumour suppressor genes.

As a first step towards identifying such genes, numerous investigators have compared the karyotypes of malignant and non-malignant hybrids in attempts to identify specific chromosomes the loss of which is associated with malignancy. Studies of intraspecies rodent cell hybrids have been hampered both by the difficulty of distinguishing the parental origin of chromosomes in the hybrids, and by the random and often rapid loss of chromosomes from the hybrids. As a result several groups have examined human × rodent cell hybrids because chromosome loss is not random (human chromosomes are preferentially lost) and because human and rodent chromosomes can be distinguished. However the rapidity with which human chromosomes are lost still provides a serious obstacle since re-expression of malignancy is usually associated with the loss of multiple chromosomes. It is therefore not surprising that both of these approaches have usually failed to identify specific suppressor chromosomes although there are a few notable exceptions to this (Klinger, 1982; Evans *et al.*, 1982; Stoler & Bouck, 1985).

Perhaps the most penetrating insights have come from the use of intraspecies human cell hybrids which have been found to exhibit a very stable karyotype. Extensive studies by both Stanbridge and his co-workers (Srivatsan *et al.*, 1986) and Klinger with his colleagues (Kaelbling & Klinger, 1986) have implicated chromosome 11 from normal human fibroblasts in the suppression of tumorigenicity of HeLa cervical carcinoma cells. These results have been confirmed by an elegant series of experiments in which the technique of microcell fusion was used to introduce a single fibroblast chromosome 11 into HeLa cells and thereby suppress tumorigenicity of the recipient cells (Saxon *et al.*, 1986). By contrast fibroblast chromosome 1 has been reported to suppress tumorigenicity of human HT 1080 fibrosarcoma cells (Benedict *et al.*, 1984). This suggests that different tumour suppressor loci may be defective in different tumour types, a possibility that is supported by the observation that HeLa × HT 1080 cell hybrids are non-tumorigenic (Weissman & Stanbridge, 1983). Stanbridge's group have also pointed out that non-tumorigenic HeLa × fibroblast hybrids remain transformed *in vitro* (Stanbridge *et al.*, 1982), a finding which accords well with the concept that malignancy is a multistep process (Klein & Klein, 1985).

### Gene loss or inactivation in tumours

Recessive mutations at specific loci are implicated in the development of a variety of human and animal tumours (Knudson, 1985). In Drosophila recessive mutations at more than 20 different loci can result in a variety of tissue-specific tumours (Gateff, 1978; Gatef, 1982) and, further up the phylogenetic scale, genetic studies of Xiphophorus, a genus of Central American fish, suggest that loss or inactivation of regulatory genes allows a proto-oncogene to promote subsequent tumour formation (Anders, 1983; Anders *et al.*, 1985). The affected genes have been referred to as anti-oncogenes (Green & Wyke, 1985; Knudson, 1985) although this term should not be taken to necessarily imply a direct interaction with oncogenes.

Retinoblastoma and Wilms' tumour are the best studied human malignancies involving recessive mechanisms. Knudson (1971) originally suggested that retinoblastomas result from two sequential mutations and it has subsequently become evident that these affect both alleles of a particular gene; in familial cases the first mutation is inherited, whereas both mutations are somatic in sporadic cases. This explains the apparent paradox whereby predisposition to the tumour may be inherited as an autosomal dominant trait at the level of the whole organism, whilst at the cellular level the neoplastic defect is recessive. This theory has been extended by cytogenetic (Francke, 1976) and linkage (Sparkes *et al.*, 1980) studies that have mapped the retinoblastoma locus (Rb1) to band q14 on chromosome 13. Restriction fragment polymorphism (RFLP) analysis has identified mechanisms by which the wild type allele is eliminated in tumours (Cavanee *et al.*, 1983): most cases appear to involve mitotic non-disjunction or recombination with the resultant loss of all or part of the chromosome carrying the normal allele (Figure 1, i–iv). Cavenee and collaborators have also shown that the tumorigenic effect of recessive mutations at the Rbl locus is not confined to the retina. Survivors of the heritable form of retinoblastoma have a greatly increased chance of developing osteosarcomas which also involve recessive changes at the Rbl locus, and similar alterations occur during the development of sporadic osteosarcomas in patients with no history of the eye tumour (Hansen *et al.*, 1985).

Wilms' tumour or nephroblastoma also occurs in heritable and sporadic forms and familial cases may be associated with aniridia, genito-urinary abnormalities and mental retardation. Cytogenetic studies of individuals with the heritable form of the disease have revealed an association with constitutional deletions involving band p13 of chromosome 11 (Riccardi *et al.*, 1978; Francke *et al.*, 1979) and similar abnormalities are seen in some tumours from patients with no family history of the disease (Slater, 1986). In tumours with an apparently normal karyotype, and in cases where no karyotypic data are available, RFLP analysis has confirmed the presence of deletions involving the short arm of chromosome 11 (Fearon *et al.*, 1984; Koufos *et al.*, 1984; Orkin *et al.*, 1984) and similar alterations have been reported to occur

**Figure 1** The use of restriction fragment length polymorphism (RFLP) analysis to study mechanisms responsible for loss/inactivation of the normal RB1 allele in retinoblastoma (Cavenee *et al.*, 1983). The cells of a patient who inherits one chromosome 13 carrying a mutant Rbl gene (rb) will also contain a normal chromosome 13 carrying a wild type Rbl gene (RB). The figure demonstrates a case in which the affected individual is heterozygous for each of 3 pairs of RFLPs (al/a2, bl/b2, and cl/c2) flanking the Rbl locus on the long arm of chromosome 13. During the development of a retinoblastoma the normal Rbl gene may be lost/inactivated by a number of different molecular mechanisms, most of which can be distinguished by the RFLP pattern present in the tumour: (i) chromosomal non-disjunction, (ii) non-disjunction followed by duplication of mutant chromosome, (iii) mitotic recombination, (iv) point mutation or gene conversion.

during the development of other embryonal tumours (Koufos *et al.*, 1985). These data are all consistent with the presence of a recessive tumour gene at 11p13. However a simple 'two hit' hypothesis would suggest that maternal and paternal alleles of such a gene would have an equal probability of being affected by each hit. One hint that the mechanisms may not be quite so simple comes from the finding that the maternal chromosome 11 is lost much more frequently than its paternal homologue in sporadic Wilms' tumours (Schroeder *et al.*, 1987). The explanation for this observation is unclear but may involve differential sensitivity of maternal and paternal gametes to mutations at 11p13 or the presence of a linked transforming gene that is differentially expressed by maternal and paternal copies of chromosome 11 (Wilkins, 1988).

Although both retinoblastoma and Wilms' tumour are rare malignancies, evidence is emerging that similar mechanisms are involved in colon cancer, a much more prevalent tumour. Once again vital clues to the location of the gene involved came from studies of a familial form of the disease, in this case familial adenomatous polyposis (FAP) in which one or more polyps almost invariably progresses to invasive carcinoma. Following a single case report of a patient with a constitutional deletion of 5q associated with adenomatous polyposis, RFLP analysis of FAP pedigrees has localised the FAP gene to 5q 21–22 (Bodmer *et al.*, 1987). Having identified this region as being of potential importance in colonic malignancy, a minisatellite probe specific for 5q was used to demonstrate loss of heterozygosity in the terminal part of 5q in 20% of spontaneous colon cancers (Solomon *et al.*, 1987).

These paradigms have stimulated much interest and there is now a rapidly growing list of human tumours associated with genetic loss at specific chromosomal locations (Table I). In the light of these observations it seems probable that gene loss/inactivation will play an important role in a very wide variety of human tumours. But what of the relationship between the genes lost or inactivated in tumours and the tumour suppressor genes detected *in vitro*? It is highly likely that they are related and preliminary evidence for this contention has recently been obtained: Stanbridge and his co-workers have shown that tumorigenicity of Wilms' tumour cells can be suppressed by the introduction of a single human chromosome 11 (Weissman *et al.*, 1987), although further experiments are needed to show that it is indeed the p13 region and not some other linked locus that mediates suppression.

### Relationship to proto-oncogenes

The evidence surveyed above suggests that various aspects of the malignant phenotype may result from loss or inactivation of specific cellular genes. However these observations need to be reconciled with the mass of evidence that proto-oncogene activation plays a central role in malignancy.

**Table I** Examples of genetic loss associated with human tumours

| *Tumour* | *Chromosomal site involved* | *Reference* |
|---|---|---|
| Retinoblastoma<br>Osteosarcoma | 13q14 | Francke, 1976; Knudson *et al.*, 1976;<br>Hansen *et al.*, 1985 |
| Wilms' tumour<br>Hepatoblastoma<br>Rhabdomyosarcoma | 11p13 | Fearon *et al.*, 1984; Koufos *et al.*, 1984;<br>Orkin *et al.*, 1984; Koufos *et al.*, 1985 |
| Bladder carcinoma | 11p | Atkin & Baker, 1984; Fearon *et al.*, 1985 |
| Bilateral acoustic neuroma | 22 | Seizinger, *et al.*, 1986; Seizinger *et al.*, 1987*a* |
| Meningioma | 22 | Seizinger *et al.*, 1987*b* |
| Myelodysplasia and acute myeloid leukaemia | 5p and 7 | Yunis, 1983 |
| 'Lymphomatous ALL' | 9p (21–22) | Chilcote *et al.*, 1985 |
| Follicular non-Hodgkin's lymphoma | 6p and 13q32 | Yunis *et al.*, 1987 |
| Small cell lung cancer | 3p (14–23) | Whang-Peng *et al.*, 1982;<br>Brauch *et al.*, 1987 |
| Renal cell carcinoma | 3p | Zbar *et al.*, 1987; Kovacs *et al.*, 1988 |
| Multiple endocrine neoplasia syndromes: | | |
| type 1 | 11 | Larsson *et al.*, 1988 |
| type 2 | 1 | Mathew *et al.*, 1987 |
| Colon cancer | 5q<br>17 | Solomon *et al.*, 1987<br>Fearon *et al.*, 1987 |
| Breast cancer | 11<br>13 | Ali *et al.*, 1987<br>Lundberg *et al.*, 1987 |

As described above the suppression of HeLa cell tumorigenicity by human fibroblasts has been studied in great detail, but unfortunately virtually nothing is known about the contribution that activated proto-oncogenes make to the malignant properties of HeLa cells. The interaction of tumour suppressor genes with proto-oncogenes has therefore been addressed by studying the suppression of malignant cells known to contain an activated proto-oncogene. Studies of the HT 1080 human fibrosarcoma cell line and the EJ human bladder cell line, which contain an activated N-*ras* and Ha-*ras* gene respectively (Hall *et al*., 1983; Parada *et al*., 1982) have produced three important observations:

1. The effect of activated *ras* genes may be suppressed at a post-translational level, perhaps by interference with the *ras* protein or its substrate. Hence suppression of EJ cell tumorigenicity by fusion with human fibroblasts is not accompanied by any alteration in the level of the protein product of the activated *ras* gene (Geiser *et al*., 1986).
2. The phenotype of cell hybrids may reflect the net effect of two opposing influences; the number of suppressor chromosomes relative to the number of chromosomes carrying activated proto-oncogenes. Thus diploid human fibroblasts can suppress the tumorigenicity of near-diploid HT 1080 cells but not that of near-tetraploid HT 1080 variants (Benedict *et al*., 1984).
3. Suppression of HT 1080 tumorigenicity is probably mediated by chromosome 1, the very chromosome to which N-*ras* has been mapped (Benedict *et al*., 1984). Chromosome 1 may therefore carry a tumour suppressor gene in addition to N-*ras* but an alternative possibility is that the unaltered N-*ras* allele itself is capable of regulating its activated counterpart.

Several investigators have utilised a different approach and studied suppression of rodent cells transformed by the introduction of an activated proto-oncogene. These experimental systems have the advantage that the activated proto-oncogenes are known to be causally related to the phenotype being studied and they have revealed two different molecular mechanisms of suppression. Both transformation and tumorigenicity of Ha-*ras* transformed cells are suppressed by fusion to untransformed cells, but suppressed hybrids thus obtained continue to express the activated Ha-*ras* gene, the effect of which is presumably inhibited at a post-translational level (Craig & Sager, 1985; Griegel *et al*., 1986). A different mechanism has been revealed by analysis of untransformed hybrids resulting from the fusion of Rous sarcoma virus-transformed rat fibroblasts (containing the v-*src* oncogene) to untransformed rodent cell lines. In this system suppression operates by repression of proviral transcription and it has also been shown that the susceptibility of a provirus to suppression is greatly influenced by its cellular integration site (Dyson *et al*., 1982). Suppression of RSV-induced transformation may therefore be mediated by a family of negative regulatory genes that control transcription within specific regions of the cellular genome (Green & Wyke, 1985; Wyke & Green, 1986). Moreover some members of such a family of regulatory genes might be expected to act as targets for carcinogenic recessive mutations (Comings, 1973).

A third strategy is to look for proto-oncogene expression in tumours known to involve loss or inactivation of specific loci but the results are difficult to interpret. Thus retinoblastomas express high levels of N-*myc* and as a result it has been suggested that one function of the Rbl gene is to repress N-*myc* expression (Lee *et al*., 1984). This now seems unlikely since the level of N-*myc* expression in retinoblastomas, although significantly higher than that found in fibroblasts or normal adult retina, is similar to the level found in normal foetal retina and, furthermore, osteosarcomas that involve inactivation of the Rbl locus do not express high levels of N-*myc* (Squire *et al*., 1986).

It is apparent that in most instances genes which are tumorigenic by virtue of loss of their function represent a novel family of tumour genes quite distinct from proto-oncogenes. However the issue is complicated by recent evidence which suggests that some proto-oncogenes may be activated by recessive mutations. Thus several tumours known to contain an activated *ras* gene have deleted the normal *ras* allele (Santos *et al*., 1982; Feinberg *et al*., 1983; Guerrero *et al*., 1985); studies of mouse skin carcinogenesis have shown that whereas papillomas contain both an activated and a normal Ha-*ras* allele, progression to invasive carcinoma may be accompanied by loss of the normal allele (Quintanilla *et al*., 1986); and the chromosome carrying an unaltered N-*ras* allele can suppress tumorigenicity of HT 1080 cells containing an activated N-*ras* gene (*vide supra*). These data emphasise that genes which are tumorigenic in a recessive mode may fall into two categories: those that are neoplastic solely by virtue of net loss of their function and proto-oncogenes which exert a neoplastic effect when loss of one allele unmasks an activating mutation in its homologue.

### Cloning recessive tumour genes

Approximately 40 proto-oncogenes have already been cloned and the relative ease with which this has been achieved reflects several factors. Many proto-oncogenes were originally identified as components of tumorigenic retroviruses thus greatly simplifying their isolation. In addition certain activated proto-oncogenes can be detected by their ability to transform cells *in vitro* or to render them tumorigenic. The ability to both recognise and select for cells that have incorporated an activated proto-oncogene has been used as the basis for the isolation of several different proto-oncogenes (Der *et al*., 1982; Hall *et al*., 1983; Fasano *et al*., 1984).

The isolation of genes that are absent or inactivated in tumours is inherently more difficult. However in a major recent advance two groups have isolated what appears to be a cDNA clone of the retinoblastoma gene by 'walking' along chromosome 13 from an anonymous sequence closely linked to the Rbl locus (Friend *et al*., 1986; Lee *et al*., 1987*a*). When used as a probe in Northern blots it detects a discrete transcript in normal retinal cells and in a variety of tumour cell lines, but not in retinoblastoma cells. Furthermore in Southern blots it reveals rearrangements and deletions in a minority of retinoblastomas and associated second tumours. That only a minority of tumours have abnormal restriction fragments is not surprising since many small structural aberrations capable of inactivating the gene will not be detected by Southern blotting. The Wilms' tumour locus is also being studied intensively (van Heyningen *et al*., 1985; Porteous *et al*., 1987) and it is anticipated that this will be characterised in the near future.

The isolation of genes involved in the hybrid suppression of malignancy is in some ways even more challenging since there are no chromosomal deletions that can be used to localise the gene and act as a starting point for cloning quests. At least two strategies are currently in use. The first involves the transfer of DNA from normal cells into transformed recipients; cells that have taken up a tumour suppressor gene can be isolated by selecting for recipient cells with a normal phenotype, and the introduced gene eventually cloned after two or more rounds of transfection. This approach is hampered by the technical difficulty of separating slow-growing and contact-inhibited untransformed cells from a population of transformed cells. However Schaefer *et al*. (1988) have overcome this problem by using ouabain to select for untransformed cells and they have recently reported the isolation of a putative human suppressor gene capable of partially reversing the transformed phenotype of *ras*-transfected rat fibroblasts. An alternative approach is based on the premise that inactivation of a tumour suppressor gene by the insertion of a retrovirus may result in transformation of a previously untransformed cell. This 'insertional mutagenesis' approach is particularly powerful since the transformed clone would be easy to isolate and the integrated provirus would provide a molecular tag for cloning the tumour suppressor gene. For this approach to be feasible the target gene must be present as a single functional copy, a situation that obtains in some cell hybrids that have retained only a single copy of the suppressor chromosome.

### Functions

Proto-oncogenes are structurally and functionally heterogenous and it is likely that recessively acting tumour genes will prove equally diverse. Indeed evidence is accumulating that, whereas proto-oncogenes apparently function in positive growth regulatory pathways, genes that are carcinogenic when their function is reduced or abolished will prove to be involved at various levels in comparable negative regulatory pathways. Several points are relevant to this hypothesis:

1. Studies of the suppression of HeLa cell tumorigenicity have shown that both tumorigenic hybrids and HeLa cells themselves rapidly produce undifferentiated tumours after injection into nude mice, whereas non-tumorigenic hybrids undergo terminal differentiation. Moreover the pathway of differentiation adopted by hybrid cells is dictated by the normal diploid parental cell. Thus fibroblast × Hela cell hybrids become fibroblastoid (Stanbridge *et al.*, 1982) whereas keratinocyte × HeLa cell hybrids differentiate into keratinising epithelium (Peehl & Stanbridge, 1982). These results are consistent with the possibility that, in this experimental system, tumour suppressor genes encode molecules which allow hybrid cells to respond to differentiation inducing signals *in vivo*.
2. The recent cloning of two recessive tumour genes (Mechler *et al.*, 1985; Friend *et al.*, 1986; Lee *et al.*, 1987*a*) has allowed direct analysis of their protein products. Mutation of the drosophilia 1(2) gl gene is implicated in larval neuroblastomas and imaginal disc tumours. Its protein product localises to the cell membrane and intercellular matrix and its expression during embryonic and larval development correlates with cessation of cellular proliferation suggesting that it may mediate proliferation arrest (Klambt & Schmidt, 1986). By contrast the retinoblastoma gene product is a nuclear phosphoprotein with DNA binding activity and so may act to regulate other cellular genes (Lee *et al.*, 1987*b*).
3. An increasing number of peptides are known to exhibit differentiation-inducing or growth-inhibiting effects (Roberts *et al.*, 1985; Marx, 1986; Beutler & Cerani, 1987; Gearing *et al.*, 1987). This hypothesis predicts that the genes encoding such inhibitory factors and their cellular receptors would be prime targets for recessive carcinogenic mutations.

### Clinical applications

It is not premature to consider the potential implications of these insights. RFLP analysis has already been used as an approach to ante-natal diagnosis in pedigrees at risk for retinoblastoma (Cavenee *et al.*, 1986; Wiggs *et al.*, 1988) and the availability of cloned probes for the locus should greatly increase predictive accuracy. Both diagnostic and prognostic information is also likely to ensue from the characterisation of other genes that are tumorigenic by virtue of their inactivation.

Possible therapeutic applications are tantalizing but distant. Antibodies which stimulate cellular receptors for inhibitory factors or the inhibitory factors themselves may prove clinically useful. It will also soon be possible to construct retroviral vectors containing genes capable of repressing the malignant phenotype and perhaps it will even prove feasible to suppress various aspects of malignancy *in vivo* by use of such vectors. As with all anti-tumour therapy, the effects are unlikely to be completely specific for tumour cells, but the potential importance of such novel therapeutic modalities remains considerable.

I am indebted to Dr J.A. Wyke, Dr C.J. Poole, Professor A. Jacobs and Dr D.P. Bentley for helpful comments on this manuscript and to Miss Nikki Pinchen and Mrs Marion Watkins for their secretarial expertise.

### References

ALI, I.U., LIDEREAU, R., THEILLET, C. & CALLAHAN, R. (1987). Reduction to homozygosity of genes on chromosome 11 in human breast neoplasia. *Science*, **238**, 185.

ANDERS, F. (1983). The biology of an oncogene, based upon studies in neoplasia in xiphophorus. *Modern Trends in Human Leukaemia V*, p. 186. Springer: Berlin.

ANDERS, F., SCHARTL, M., BARNEKOW, A. & 4 others (1985). The genes that carcinogens act upon. Haematology and blood transfusion, Vol. 29, *Modern Trends in Human Leukaemia VI*, Neth, Gallo, Greaves, Janka (eds) p. 228. Springer-Verlag: Berlin.

ATKIN, N.B. & BAKER, M.C. (1984). Cytogenetic study of ten carcinomas of the bladder: involvement of chromosomes 1 and 11. *Cancer Genet. Cytogenet.*, **15**, 253.

BENEDICT, W.F., WEISSMAN, B.E., MARK, C. & STANBRIDGE, E.J. (1984). Tumorigenicity of human HT 1080 fibrosarcoma × normal fibroblast hybrids: chromosome dosage dependency. *Cancer Res.*, **44**, 3471.

BEUTLER, B. & CERAMI, A. (1987). Cachectin: more than a tumor necrosis factor. *N. Engl. J. Med.*, **316**, 379.

BISHOP, J.M. (1981). Enemies within: the genesis of retrovirus oncogenes. *Cell*, **23**, 5.

BISHOP, J.M. (1987). The molecular genetics of cancer. *Science*, **235**, 305.

BODMER, W.F., BAILEY, C.J. BODMER, J. & 10 others (1987). Localisation of the gene for familial adenomatous polyposis on chromosome 5. *Nature*, **328**, 614.

BOS, J.L., FEARON, E.R., HAMILTON, S.R. & 4 others (1987). Prevalence of *ras* gene mutations in human colorectal cancers. *Nature*, **327**, 293.

BOVERI, T. (1914). Zur frage der Entstehung Malinger Tumoren. Jena, Germany, Fischer.

BRAUCH, H., JOHNSON, B. HOVIS, J. & 9 others (1987). Molecular analysis of the short arm of chromosome 3 in small-cell and non-small-cell carcinoma of the lung. *N. Engl. J. Med.*, **317**, 1109.

CAVENEE, W.K., DRYJA, T.P., PHILLIPS, R.A. & 6 others (1983). Expression of recessive alleles by chromosomal mechanisms in retinoblastoma. *Nature*, **305**, 779.

CAVENEE, W.K., MURPHEE, A.L., SHULL, M.M. & 4 others (1986). Prediction of familial predisposition to retinoblastoma. *N. Engl. J. Med.*, **314**, 1201.

CHAMPLIN, R.E. & GOLDE, D.W. (1985). Chronic myelogenous leukaemia: recent advances. *Blood*, **65**, 1039.

CHAN, J.Y.H., BECKER, F.F., GERMAN, J. & RAY, J.H. (1987). Altered DNA ligase I activity in Bloom's syndrome cells. *Nature*, **325**, 357.

CHILCOTE, R.R., BROWN, E. & ROWLEY, J.D. (1985). Lymphoblastic leukemia with lymphomatous features associated with abnormalities of the short arm of chromosome 9. *N. Engl. J. Med.*, **313**, 286.

CLEAVER, J.E. (1968). Defective repair replication of DNA in xeroderma pigmentosum. *Nature*, **218**, 652.

COMINGS, D.E. (1973). A general theory of carcinogenesis. *Proc. Natl Acad. Sci. USA*, **70**, 3324.

CORY, S. (1986). Activation of cellular oncogenes in hemopoietic cells by chromosome translocations. *Adv. Cancer Res.*, **47**, 189.

CRAIG, R.W. & SAGER, R. (1985). Suppression of tumorigenicity in hybrids of normal and oncogene-transformed CHEF cells. *Proc. Natl Acad. Sci. USA*, **82**, 2062.

DAY, R.S., ZIOLKOWSKI, C.H.J., SCUDIERO, D.A. & 5 others (1980). Defective repair of alkylated DNA by human tumour and SV40-transformed human cell strains. *Nature*, **288**, 724.

DER, C.J., KRONTIRIS, T.G. & COOPER, G.M. (1982). Transforming genes of human bladder and lung carcinoma cell lines are homologous to the *ras* genes of Harvey and Kirsten sarcoma viruses. *Proc. Natl Acad. Sci. USA*, **79**, 3637.

DYSON, P.J., QUADE, K. & WYKE, J.A. (1982). Expression of the ASV src gene in hybrids between normal and virally transformed cells: specific suppression occurs in some hybrids but not others. *Cell*, **30**, 491.

EDITORIAL (1986). Growth factors and malignancy. *Lancet*, **II**, 317.

EDITORIAL (1987). Gene amplification and malignancy. *Lancet*, **I**, 839.

EVANS, E.P., BURTENSHAW, M.D., BROWN, B.B., HENNION, R. & HARRIS, H. (1982). The analysis of malignancy by cell fusion IX. Re-examination and clarification of the cytogenetic problem. *J. Cell Sci.*, **56**, 113.

FASANO, O., BIRNBAUM, D., EDLUND, L., FOGH, J. & WIGLER, M. (1984). New human transforming genes detected by a tumorigenicity assay. *Mol. Cell. Biol.*, **4**, 1695.

FEARON, E.R., VOGELSTEIN, B. & FEINBERG, A.P. (1984). Somatic deletion and duplication of genes on chromosome 11 in Wilms' tumour. *Nature*, **309**, 176.

FEARON, E.R., FEINBERG, A.P., HAMILTON, S.H. & VOGELSTEIN, B. (1985). Loss of genes on the short arm of chromosome 11 in bladder cancer. *Nature*, **318**, 377.

FEARON, E.R., HAMILTON, S.H. & VOGELSTEIN, B. (1987). Clonal analysis of human colorectal tumours. *Science*, **238**, 193.

FEINBERG, A.P., VOGELSTEIN, B., DROLLER, M.J., BAYLIN, S.B. & NELKIN, B.D. (1983). Mutation affecting the 12th amino acid of the c-Ha-*ras* oncogene product occurs infrequently in human cancer. *Science*, **220**, 1175.

FRANCKE, U. (1976). Retinoblastoma and chromosome 13. *Cytogenet. Cell Genet.*, **14**, 131.

FRANCKE, U., HOMES, L.B., ATKINS, L. & RICCARDI, V.M. (1979). Aniridia–Wilms' tumour association: evidence for specific deletions of 11p13. *Cytogenet. Cell Genet.*, **24**, 185.

FRIEND, S.H., BERNARDS, R., ROGELJ, S. & 4 others. (1986). A human DNA segment with properties of the gene that predisposes to retinoblastoma and osteosarcoma. *Nature*, **323**, 643.

GATEFF, E., (1978). Malignant neoplasms of genetic origin in *Drosophila melanogaster*. *Science*, **200**, 1448.

GATEFF, E. (1982). Cancer, genes and development: the Drosophila case. *Adv. Cancer Res.*, **37**, 33.

GEARING, D.P., GOUGH, N.M., KUNG, J.A. & 6 others (1987). Molecular cloning and expression of cDNA encoding a murine myeloid leukemia inhibitory factor (LIF). *EMBO. J.*, **6**, 3995.

GEISER, A.G., DER, C.J., MARSHALL, C.J. & STANBRIDGE, E.J. (1986). Suppression of tumorigenicity with continued expression of the c-Ha-*ras* oncogene in EJ bladder carcinoma–human fibroblast hybrid cells. *Proc. Natl Acad. Sci. USA*, **83**, 5209.

GREEN, A.R. & WYKE, J.A. (1985). Anti-oncogenes. A subset of regulatory genes involved in carcinogenesis? *Lancet*, **II**, 475.

GRIEGEL, S., TRAUB, O., WILLECKE, E. & SCHAFER, R. (1986). Suppression and re-expression of transformed phenotype in hybrids of Ha-ras-1 transformed rat-1 cells and early passage rat embryonic fibroblasts. *Int. J. Cancer*, **38**, 697.

GUERRERO, I., VILLASANTE, A., CORCES, V. & PELLICER, A. (1985). Loss of the normal N-*ras* allele in a mouse thymic lymphoma induced by a chemical carcinogen. *Proc. Natl Acad. Sci. USA*, **82**, 7810.

HALL, A., MARSHALL, C.J., SPURR, N.K. & WEISS, R.A. (1983). Identification of the transforming gene in two human sarcoma cell lines as a new member of the *ras* gene family located on chromosome 1. *Nature*, **303**, 396.

HANSEN, M.F., KOUFOS, A., GALLIE, B.L. & 5 others (1985). Osteosarcoma and retinoblastoma: a shared chromosomal mechanism revealing recessive predisposition. *Proc. Natl Acad. Sci. USA*, **82**, 6216.

HARRIS, H., MILLER, O.J., KLEIN, G., WORST, P. & TACHIBANA, T. (1969). Suppression of malignancy by cell fusion. *Nature*, **223**, 363.

KAELBLING, M. & KLINGER, H.P. (1986). Suppression of tumorigenicity in somatic cell hybrids III Cosegregation of human chromosome 11 of a normal cell and suppression of tumorigenicity in intra species hybrids of normal diploid × malignant cells. *Cytogenet. Cell Genet.*, **41**, 65.

KAELBLING, M., REGINSKI, R.S. & KLINGER, H.P. (1986). DNA polymorphisms indicate loss of heterozygosity for chromosome 11 of D98AH2 cells. *Cytogenet. Cell Genet.*, **41**, 240.

KAHN, P., LEUTZ, A. & GRAF, T. (1986). Individual and combined effects of viral oncogenes in hematopoietic cells. In *Oncogenes and Growth Control*, Kahn, P. and Graf, T. (eds) p. 312. Springer-Verlag.

KLAMBT, C. & SCHMIDT, O. (1986). Developmental expression and tissue distribution of the *lethal* (2) *giant larvae* protein of *Drosophila melanogaster*. *EMBO. J.*, **5**, 2955.

KLEIN, G. & KLEIN, E. (1985). Evolution of tumours and the impact of molecular oncology. *Nature*, **315**, 190.

KLEIN, G., BREGULA, U., WEINER, F. & HARRIS, H. (1971). The analysis of malignancy by cell fusion. I. Hybrids between tumour cells and L cell derivatives. *J. Cell Sci.*, **8**, 659.

KLINGER, H.P. (1982). Suppression of tumorigenicity. *Cytogenet. Cell Genet.*, **32**, 68.

KNUDSON, A.G. (1971). Mutation and cancer: statistical study of retinoblastoma. *Proc. Natl Acad. Sci. USA*, **68**, 820.

KNUDSON, A.G. (1985). Hereditary cancer, oncogenes and anti-oncogenes. *Cancer Res.*, **45**, 1437.

KNUDSON, A.G., MEADOWS, A.T., NICHOLS, W.W. & HILL, R. (1976). Chromosomal deletion and retinoblastoma. *N. Engl. J. Med.*, **295**, 1120.

KOUFOS, A., HANSEN, M.F., LAMPKIN, B.C. & 4 others (1984). Loss of alleles at loci on human chromosome 11 during genesis of Wilms' tumour. *Nature*, **309**, 170.

KOUFOS, A., HANSEN, M.F., COPELAND, N.G., JENKINS, N.A., LAMPKIN, B.C. & CAVENEE, W.K. (1985). Loss of heterozygosity in three embryonal tumours suggests a common pathogenetic mechanism. *Nature*, **316**, 330.

KOVACS, G., ERLANDSSON, R., BOLDOG, F. & 4 others (1988). Consistent chromosome 3p deletion and loss of heterozygosity in renal cell carcinoma. *Proc. Natl Acad. Sci. USA*, **85**, 1571.

LAND, H., PARADA, L.F. & WEINBERG, R.A. (1983). Tumorigenic conversion of primary embryo fibroblasts requires at least two cooperating oncogenes. *Nature*, **304**, 596.

LAND, H., CHEN, A.C., MORGENSTERN, J.P., PARADA, L.F. & WEINBERG, R.A. (1986). Behaviour of *myc* and *ras* oncogenes in transformation of rat embryo fibroblasts. *Mol. Cell. Biol.*, **6**, 1917.

LARSSON, C., SKOGSEID, B., OBERG, E., NAKAMURA, Y. & NORDERSKJOLD, M. (1988). Multiple endocrine neoplasia type 1 gene maps to chromosome 11 and is lost in insulinoma. *Nature*, **332**, 85.

LEE, W.H., MURPHEE, A.L. & BENEDICT, W.F. (1984). Expression and amplification of the N-*myc* gene in primary retinoblastoma. *Nature*, **309**, 458.

LEE, W., BOOKSTEIN, R., HONG, F., YOUNG, L., SHEW. J. & LEE, E. (1987*a*). Human retinoblastoma susceptibility gene: cloning, identification and sequence. *Science*, **235**, 1394.

LEE, W.H., SHEW, J.-Y., HONG, F.D. & 5 others (1987*b*). The retinoblastoma susceptibility gene encodes a nuclear phosphoprotein associated with DNA binding activity. *Nature*, **329**, 642.

LEHMAN, A.R. (1982). Xeroderma pigmentosum, Cockayne syndrome and ataxia-telangiectasia: disorders relating DNA repair to carcinogenesis. *Cancer Suveys*, **1**, 93.

LUNDBERG, C., SKOOG, L., CAVENEE, W.K. & NORDENSKJOLD, M. (1987). Loss of heterozygosity in human ductal breast tumors indicates a recessive mutation on chromosome 13. *Proc. Natl Acad. Sci. USA*, **84**, 2372.

MARX, J.L. (1986). The yin and yang of cell growth control. *Science*, **232**, 1093.

MATHEW, C.G.P., SMITH, B.A., THORPE, K. & 4 others (1987). Deletion of genes on chromosome 1 in endocrine neoplasia. *Nature*, **328**, 524.

MECHLER, B.M., McGINNIS, W. & GEHRING, W.J. (1985). Recessive oncogene of *Drosophila melanogaster*. *EMBO. J.*, **4**, 1551.

MILLER, E.C. (1978). Some current perspectives on chemical carcinogenesis in humans and experimental animals: presidential address. *Cancer Research*, **38**, 1479.

MILLER, D.A. & MILLER, O.J. (1983). Chromosomes and cancer in the mouse: studies in tumors, established cell lines and cell hybrids. *Adv. Cancer Res.*, **39**, 153.

OHNO, S. (1971). Genetic implication of karyological instability of malignant somatic cells. *Physiological Reviews*, **51**, 496.

ORKIN, S.H., GOLDMAN, D.S. & SALLAN, S.E. (1984). Development of homozygosity for chromosome 11p markers in Wilms' tumour. *Nature*, **309**, 172.

PARADA, L.F., TABIN, C.F., SHIH, C. & WEINBERG, R.A. (1982). Human EJ bladder carcinoma oncogene is homologue of Harvey sarcoma virus *ras* gene. *Nature*, **297**, 474.

PATERSON, M.C., SMITH, B.P., LOHMAN, P.H.M., ANDERSON, A.K. & FISHMAN, L. (1976). Defective excision repair of X-ray damaged DNA in human (ataxia telangiectasia) fibroblasts. *Nature*, **260**, 444.

PEEHL, D.M. & STANBRIDGE, E.J. (1982). The role of differentiation in the control of tumorigenic expression in human cell hybrids. *Int. J. Cancer*, **30**, 113.

PORTEOUS, D.J., BICKMORE, W., CHRISTIE, S. *et al.* (1987). HRAS 1-selected chromosome transfer generates markers that colocalise aniridia and genitourinary dysplasia-associated translocation breakpoints and the Wilms' tumor gene within band 11p13. *Proc. Natl Acad. Sci. USA*, **84**, 5355.

QUINTANILLA, M., BROWN, K., RAMSDEN, M. & BALMAIN, A. (1986). Carcinogen-specific mutation and amplification of Ha-*ras* during mouse skin carcinogenesis. *Nature*, **322**, 78.

RICCARDI, V.M., SUJANSKY, E., SMITH, A.C. & FRANCKE, U. (1978). Chromosomal imbalance in the aniridia–Wilms' tumor association: 11p interstitial deletion. *Pediatrics*, **61**, 604.

ROBERTS, A.B., ANZANO, M.A., WAKEFIELD, L.M., ROCHE, N.S., STERN, D.F. & SPORN, M.B. (1985). Type B transforming growth factor: a bifunctional regulator of cellular growth. *Proc. Natl Acad. Sci. USA*, **82**, 119.

SAGER, R. (1985). Genetic suppression of tumour formation. *Adv. Cancer Res.*, **44**, 43.

SANTOS, E., TRONICK, S.R., AARONSON, S.A., PULCIANI, S. & BARBACID, M. (1982). T24 human bladder carcinoma oncogene is an activated form of the normal human homologue of BALB- and Harvey-MSV transforming genes. *Nature*, **298**, 343.

SAXON, P.J., SRIVATSAN, E.S. & STANBRIDGE, E.J. (1986). Introduction of human chromosome 11 via microcell transfer controls tumorigenic expression of HeLa cells. *EMBO. J.*, **5**, 3461.

SCHAEFER, R., IYER, J., HEN, E. & NIRKKO, A.C. (1988). Partial reversion of the transformed phenotype in HRAS-transfected tumorigenic cells by transfer of a human gene. *Proc. Natl Acad. Sci. USA*, **85**, 1590.

SCHROEDER, W.T., CHAO, L.-Y., DAO, D.D. & 5 others (1987). Nonrandom loss of maternal chromosome 11 alleles in Wilms' tumors. *Am. J. Hum. Genet.*, **40**, 413.

SEEGER, R.C., BRODEUR, G.M., SATHER, H. & 4 others. (1985). Association of multiple copies of the N-*myc* oncogene with rapid progression of neuroblastoma. *N. Engl. J. Med.*, **313**, 1111.

SEIZINGER, B.R., MARTUZA, R.L. & GUSELLA, J.F. (1986). Loss of genes on chromosome 22 in tumorigenesis of human acoustic neuroma. *Nature*, **322**, 644.

SEIZINGER, B.R., ROULEAU, G., OZELIUS, L.J. & 5 others (1987*a*). Common pathogenic mechanism for three tumor types in bilateral acoustic neurofibromatosis. *Science*, **236**, 317.

SEIZINGER, B.R., DE LA MONTE, S., ATKINS, L., GUSELLA, J.F. & MARTUZA, R.L. (1987*b*). Molecular genetic approach to human meningioma: loss of genes of chromosome 22. *Proc. Natl Acad. Sci. USA*, **84**, 5419.

SLAMON, D.J., CLARK, G.M., WONG, S.G., LEVIN, W.J., ULLRICH, A. & MCGUIRE, W.L. (1987). Human breast cancer: correlation of relapse and survival with amplification of the HER-2/neu oncogene. *Science*, **235**, 177.

SLATER, R.M. (1986). The cytogenetics of Wilms' tumour. *Cancer Genet. Cytogenet.*, **19**, 37.

SOLOMON, E., VOSS, R., HALL, V. & 6 others (1987). Chromosome 5 allele loss in human colorectal carcinomas. *Nature*, **328**, 616.

SPARKES, R.S., SPARKES, M.C., WILSON, M.G. & 4 others (1980). Regional assignment of genes for human esterase D and retinoblastoma to chromosome band 13q14. *Science*, **219**, 971.

SQUIRE, J., GODDARD, A.D., CANTON, M., BECKER, A., PHILLIPS, R.A. & GALLIE, B.L. (1986). Tumour induction by the retinoblastoma mutation is independent of N-*myc* expression. *Nature*, **322**, 555.

SRIVATSAN, E.S., BENEDICT, W.F. & STANBRIDGE, E.J. (1986). Implication of chromosome 11 in the suppression of neoplastic expression in human cell hybrids. *Cancer Res.*, **46**, 6174.

STANBRIDGE, E.J. (1987). Genetic regulation of tumorigenic expression in somatic cell hybrids. *Adv. Viral Oncol.*, **6**, 83.

STANBRIDGE, E.J. & CEREDIG, R. (1981). Growth regulatory control of human cell hybrids in nude mice. *Cancer Res.*, **41**, 573.

STANBRIDGE, E.J., DER, C.J., DOERSEN, C.J. & 4 others (1982). Human cell hybrids: analysis of transformation and tumorigenicity. *Science*, **215**, 252.

STARK, G.R. (1986). DNA amplification in drug resistant cells and in tumours. *Cancer Surveys*, **5**, 1.

STOLER, A. & BOUCK, N. (1985). Identification of a single human chromosome in the normal human genome essential for suppression of hamster cell transformation. *Proc. Natl Acad. Sci. USA*, **80**, 570.

STORER, J.B. (1982). Radiation carcinogenesis. In *Cancer: A Comprehensive Treatise*, 2nd edition, vol. 1, Becker, F.F. (ed) p. 629.

SWIFT, M. (1982). In ataxia telangectasia – a cellular link between cancer, neuropathology and immune deficiency. Bridges, B.A. & Harnden, D.G. (eds) p. 355.

SWIFT, M., REITNAUER, P.J., MORRELL, D. & CHASE, C.L. (1987). Breast and other cancers in families with ataxia-talangiectasia. *N. Engl. J. Med.*, **316**, 1289.

VAN HEYNINGEN, V., BOYD, P.A., SEAWRIGHT, A. & 8 others (1985). Molecular analysis of chromosome 11 deletion in aniridia–Wilms' tumor syndrome. *Proc. Natl Acad. Sci. USA*, **82**, 8592.

WEISSMAN, B.E. & STANBRIDGE, E.J. (1983). Complementation of the tumorigenic phenotype in human cell hybrids. *J. Natl Cancer Inst.*, **70**, 667.

WEISSMAN, B.E., SAXON, P.J., PASQUALE, S.R., JONES, G.R., GEISER, A.G. & STANBRIDGE, E.J. (1987). Introduction of a normal human chromosome 11 into a Wilms' tumor cell line controls its tumorigenic expression. *Science*, **236**, 175.

WHANG-PENG, J., KAO-SHAN, C.J., LEE, E.C. & 4 others (1982). Specific chromosome defect associated with human small cell lung cancer: deletion 3p(14-23). *Science*, **215**, 181.

WIGGS, J., NORDENSKJOLD, M., YANDELL, D. & 11 others (1988). Prediction of the risk of hereditary retinoblastoma using DNA polymorphisms within the retinoblastoma gene. *N. Engl. J. Med.*, **318**, 151.

WILKINS, R.J. (1988). Genomic imprinting and carcinogenesis. *Lancet*, **I**, 329.

WILLIS, A.E. & LINDAHL, T. (1987). DNA ligase I deficiency in Bloom's syndrome. *Nature*, **325**, 355.

WYKE, J.A. & GREEN, A.R. (1986). Suppression of the neoplastic phenotype. In *Oncogenes and Growth Control*, Kahn, P. & Graf, T. (eds) p. 340. Springer-Verlag: Berlin, Heidelberg.

YUNIS, J.J. (1983). The chromosomal basis of human neoplasia. *Science*, **221**, 227.

YUNIS, J.J., FRIZZERA, G., OKEN, M.M., MCKENNA, J., THEOLOGIDES, A. & ARNESEN, M. (1987). Multiple recurrent genomic defects in follicular lymphoma. A possible model for cancer. *N. Engl. J. Med.*, **316**, 79.

ZAJAC-KAYE, M. & TS'O, P.O.P. (1984). DNAse I encapsulated in liposomes can induce neoplastic transformation of Syrian hamster embryo cells in culture. *Cell*, **39**, 427.

ZBAR, B., BRAUCH, H., TALMADGE, C. & LINEHAN, M. (1987). Loss of alleles of loci on the short arm of chromosome 3 in renal cell carcinoma. *Nature*, **327**. 721.